# Importance of Comprehensive Assessment in Brugada Syndrome. Reply to Kataoka, N.; Imamura, T. How to Diagnose and Risk Stratify Brugada Syndrome. Comment on “Matusik et al. Twelve-Lead ECG, Holter Monitoring Parameters, and Genetic Testing in Brugada Syndrome: Insights from Analysis of Multigenerational Family with a History of Sudden Cardiac Arrest during Physical Activity. *J. Clin. Med.* 2023, *12*, 6581”

**DOI:** 10.3390/jcm13030811

**Published:** 2024-01-31

**Authors:** Paweł T. Matusik, Piotr Bijak, Magdalena Kaźnica-Wiatr, Marek Karpiński, Patrycja S. Matusik, Andrzej Maziarz, Piotr Podolec, Jacek Lelakowski

**Affiliations:** 1 Institute of Cardiology, Faculty of Medicine, Jagiellonian University Medical College, 31-202 Kraków, Poland; 2 Department of Electrocardiology, St. John Paul II Hospital, 31-202 Kraków, Poland; 3 Cardiology Outpatient Clinic, St. John Paul II Hospital, 31-202 Kraków, Poland; 4 Department of Cardiac and Vascular Diseases, St. John Paul II Hospital, 31-202 Kraków, Poland; 5 Genetic Counselling Outpatient Clinic, St. John Paul II Hospital, 31-202 Kraków, Poland; 6 Department of Diagnostic Imaging, University Hospital, 30-688 Kraków, Poland; 7 Department of Radiology, Jagiellonian University Medical College, 31-501 Kraków, Poland

We would like to thank Dr. Kataoka and Dr. Imamura for their interest in our study and their valuable comments on diagnostics and risk stratification in Brugada syndrome (BrS) [1]. We are glad to have the possibility to discuss further the issues raised in our paper [2].

We consider the detailed and comprehensive assessment of patients suspected of having BrS (especially in the case of familial occurrence of the disease) to be a very important step before clinical counseling and management. The proper identification of BrS among family members (especially first-degree relatives who consist of a fairly homogenous group of patients) may reduce the risk of mental distress associated with inappropriate BrS diagnosis and unfavorable clinical outcomes, including sudden cardiac death, due to the introduction of simple lifestyle changes with or without the implantable cardioverter–defibrillator placement in affected individuals [3]. We aimed to apply and master already available tools in the current clinical practice and have shown a good prognosis in asymptomatic patients. 

In our study, we tested a panel of genes implicated in BrS using next-generation sequencing (NGS) technology, identifying a novel mutation in the sodium voltage-gated channel alpha subunit 5 (SCN5A) [2]. As mentioned in our paper, we completely agree with Dr. Kataoka and Dr. Imamura that majority of BrS patients do not have an SCN5A mutation [2]. However, according to the most recent European Society of Cardiology guidelines for the management of patients with ventricular arrhythmias and the prevention of sudden cardiac death, “genetic testing for SCN5A gene is recommended for probands with BrS” and “when a class IV or class V variant has been identified in a living or deceased individual with a condition that carries a risk of ventricular arrhythmia and sudden cardiac death, genetic testing of first-degree and symptomatic relatives and obligate carriers is recommended” (both class I, level of evidence C recommendations) [3]. Moreover, the Expert Consensus Statement on the State of Genetic Testing for Cardiac Diseases, endorsed by major international societies focusing on heart rhythm disorders, recommends genetic testing with SCN5A sequencing in patients with spontaneous type 1 BrS electrocardiographic (ECG) changes or induced during a sodium channel blocker test type 1 ECG when additional clinical features or family history are observed [4]. This document recommends variant-specific testing in family members and proper relatives when a disease-causative variant is identified [4]. Importantly, a functionally proven loss-of-function SCN5A mutation was previously shown to be associated with a worse prognosis related to an earlier occurrence of lethal arrhythmic events [5]. 

At the same time, we agree that it is crucial to distinguish diagnostics and risk stratification. However, we would like to highlight that there are diagnostic features or scores that possess prognostic meaning, including the Shanghai score (initially developed as a diagnostic score) [6]. On the other hand, some prognostic factors may increase the probability of a BrS diagnosis among family members, as suggested by our study using a positive aVR sign (reflecting the delayed onset of the right ventricular depolarization in lead aVR) [2,7,8]. In our patients, we observed several risk features identified in previous larger studies. However, at least in part due to the limited number of patients included in our study, we were unable to prospectively confirm their importance for risk stratification.

We are familiar with studies indicating features of cardiomyopathy (and also differentiated our patients with arrhythmogenic cardiomyopathy) in patients with BrS, including a systematic review on this topic [2,9]. There are several hypotheses on pathogenetic pathways implicated in BrS postulated, which were discussed by us in our manuscript as well as in our previous review paper that also focused on these aspects, and we agree that the pathogenesis of BrS is at least in part multifactorial [2,10].

Further, extensive studies on BrS are needed, which we hope will help us better understand the complex etiology of BrS, improve both BrS diagnostics and risk stratification as well as potentially enable the discovery of new personalized therapies.
